# Proteomic Stability and Ex Vivo Compatibility of a Processed Phospholipoproteic Secretome-Derived Formulation

**DOI:** 10.3390/pharmaceutics18070847

**Published:** 2026-07-12

**Authors:** Ramón Gutiérrez-Sandoval, Francisco Gutiérrez-Castro, Natalia Muñoz-Godoy, Ider Rivadeneira, Andy Lagos, Jordan Iturra, Francisco Krakowiak, Ignacio Muñoz, Andrés Toledo

**Affiliations:** 1 OGRD Alliance LLC, 6900 Tavistock Lakes Boulevard, Suite 400, Lake Nona, Orlando, FL 32827, USA; 2 Department of Cancer Research, Flowinmunocell-Bioexocell Group, 08028 Barcelona, Spain; servicios@flowinmunocell.cl (F.G.-C.); contacto@flowinmunocell.cl (N.M.-G.); 3 Department of Outreach and Engagement Programs, OGRD Consortium, Charlestown KN0802, Saint Kitts and Nevis; iderlautaro@gmail.com (I.R.); lagosandy@gmail.com (A.L.); jiconsultant@ogrdconsorcio.com (J.I.); kinesiologo@recell.cl (I.M.); 4 Histopathology Laboratory, Bioclas, Concepción 4030000, Chile; fkconsultant@ogrdconsorcio.com

**Keywords:** phospholipoproteic secretome-derived formulation, label-free quantitative proteomics, dia-PASEF, timsTOF Pro, processing stability, cryopreservation, lyophilization, live-cell kinetic imaging, short-term ex vivo compatibility, extracellular lipid–protein preparations

## Abstract

**Background**: Processed extracellular lipid–protein preparations require rigorous analytical characterization to determine whether their compositional profile, processing stability, and short-term cellular compatibility are preserved across storage and handling conditions. **Methods**: In this study, we quantitatively characterized a processed phospholipoproteic secretome-derived formulation under fresh, concentrated, cryopreserved, and lyophilized conditions. **Results**: Label-free quantitative proteomic analyses performed using timsTOF Pro mass spectrometry coupled to dia-PASEF acquisition identified 574 human proteins across all experimental conditions following predefined analytical quality criteria. Comparative analyses demonstrated preservation of the overall structural proteomic profile following processing and storage procedures, with retention of membrane-associated and extracellular structural proteins consistently exceeding 90% relative to the fresh reference condition. Quantitative reproducibility remained high across all experimental groups, with coefficients of variation ranging from 3.0% to 4.5% and strong inter-replicate Pearson correlations. Principal component analysis, hierarchical clustering, peptide/protein overlap analyses, and differential expression profiling demonstrated limited proteomic divergence while preserving the majority of quantified proteins within conserved abundance ranges. Complementary real-time live-cell kinetic imaging performed in non-malignant dermal-derived cells using the IncuCyte^®^ S3 platform demonstrated stable short-term confluence kinetics and cellular viability exceeding 92% over 48 h across all evaluated formulations. No sustained proliferative suppression or detectable morphological evidence of cytotoxicity was observed. Collectively, these findings support the preservation of compositional stability, analytical reproducibility, and short-term ex vivo cellular compatibility across defined processing and storage conditions. These integrated proteomic and kinetic datasets provide a quantitative framework for the analytical evaluation of processed extracellular phospholipoproteic preparations, while functional barrier activity, membrane incorporation, lipid raft engagement, and long-term tissue-level effects remain to be addressed in dedicated future studies.

## 1. Introduction

The plasma membrane of dermal cells contains dynamic lipid–protein microdomains enriched in cholesterol, sphingomyelin, and glycosphingolipids, commonly referred to as lipid rafts, which coexist in liquid-ordered (Lo) and liquid-disordered (Ld) phases [1]. These nanoscale membrane assemblies contribute to essential biophysical properties, including membrane fluidity, lateral protein diffusion, receptor clustering, and spatial organization of signaling platforms [2].

Within the dermal microenvironment, fibroblasts, keratinocytes, and endothelial cells are continuously exposed to oxidative stress, extracellular matrix remodeling, inflammatory mediators, and age-associated biomechanical alterations. These factors may affect membrane-associated organization and extracellular structural homeostasis, contributing to impaired barrier function, altered cellular communication, and disruption of tissue integrity in several dermatological contexts [3,4].

Secretome-derived extracellular preparations contain complex mixtures of soluble factors, extracellular structural proteins, membrane-associated proteins, lipid-associated components, and particulate extracellular fractions. Because these systems are compositionally heterogeneous, their scientific evaluation requires analytical approaches that can define protein identity, reproducibility, and stability across processing conditions before any functional interpretation is proposed. In this context, processed phospholipoproteic secretome-derived preparations represent extracellular lipid–protein systems whose characterization should be based first on compositional, proteomic, and ex vivo compatibility data rather than on presumed biological activity [5,6].

Previous studies on extracellular vesicle-related preparations, secretome-derived products, and lipid–protein extracellular assemblies have highlighted the importance of cargo heterogeneity, protein composition, processing reproducibility, and storage stability. However, substantial analytical challenges remain, particularly regarding the preservation of proteomic profiles after concentration, cryopreservation, and lyophilization. These procedures may selectively alter protein abundance, affect extracellular structural components, or modify the relative representation of membrane-associated proteins. Therefore, quantitative proteomic characterization is necessary to determine whether processed extracellular phospholipoproteic preparations retain a conserved molecular profile after handling and storage procedures [7].

**Figure 1:**
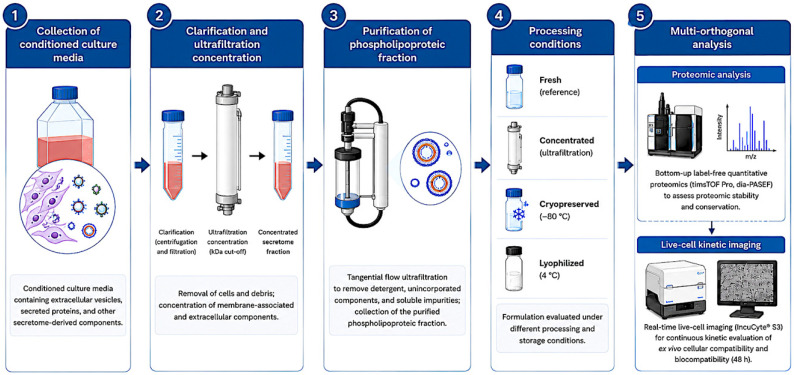
Workflow for the preparation, processing, and analytical evaluation of the processed phospholipoproteic secretome-derived formulation. Conditioned culture media containing extracellular particulate fractions, membrane-associated proteins, and extracellular structural components were subjected to sequential processing, enrichment, concentration by ultrafiltration, and purification of the phospholipoproteic fraction. The resulting formulation was evaluated under fresh, concentrated, cryopreserved, and lyophilized conditions using label-free quantitative proteomics and real-time live-cell kinetic imaging. The workflow summarizes the integrated analytical strategy used to assess compositional stability, proteomic conservation, and short-term ex vivo cellular compatibility across processing conditions. The circular schematic icons represent extracellular phospholipoproteic lipid–protein assemblies and are illustrative only; they do not indicate absolute particle size, concentration, or quantitative molecular composition.

An important consideration for secretome-derived phospholipoproteic preparations is the preservation of compositional stability during defined processing and storage procedures, including concentration, cryopreservation, and lyophilization. Maintenance of a reproducible proteomic profile across these conditions is relevant for comparative analytical characterization, quality control, and future experimental evaluation of extracellular lipid–protein preparations. The overall preparation, processing, and analytical workflow used in the present study is summarized in Figure 1.

In the present study, we quantitatively characterized a processed phospholipoproteic secretome-derived formulation enriched in extracellular lipid–protein and membrane-associated structural components across multiple processing conditions. Proteomic conservation, analytical reproducibility, and short-term ex vivo cellular compatibility were evaluated under fresh, concentrated, cryopreserved at −80 °C, and lyophilized conditions using label-free quantitative proteomics and real-time live-cell kinetic imaging in non-malignant dermal-derived cells.

Comparative analyses included principal component analysis, correlation matrices, coefficient of variation distributions, hierarchical clustering, peptide/protein overlap analyses, and differential expression profiling, as detailed in the corresponding figures and supplementary materials below. The objective of this study was to establish a quantitative analytical framework for evaluating compositional stability and short-term ex vivo compatibility of processed phospholipoproteic secretome-derived preparations.

The study was not designed to demonstrate functional skin barrier activity, membrane incorporation, lipid raft localization, or long-term tissue-level effects, which remain subjects for dedicated future investigation [8].

## 2. Materials and Methods

### 2.1. Preparation of the Processed Phospholipoproteic Secretome-Derived Formulation

The phospholipoproteic formulation evaluated in this study was obtained from processed cell-culture secretome preparations generated under controlled laboratory conditions [9]. The established human cell cultures used to generate the secretome-derived preparations corresponded to research-grade human adipose-derived mesenchymal stromal cell cultures derived from a GMP-manufactured master cell bank, internal study culture code FIC-BX-hADMSC-PSF-2026-01, provided by Flowinmunocell-Bioexocell Group, Barcelona, Spain, for research use only and maintained under controlled laboratory conditions. The source material consisted of secretome preparations derived from established human cell cultures and was used exclusively for ex vivo analytical and formulation-characterization purposes. No new human biological samples were collected for the purposes of this study, no intervention involving human participants was performed, no identifiable donor information was accessed, and no associated identifiable clinical data were used. The cellular material used to generate the secretome-derived preparations was not generated or genetically modified by the authors for the purposes of this work, and no novel human cell line was created. No animal-derived cellular material was used for the generation of the formulation evaluated in this study. Source material was handled under standardized laboratory biosafety and quality-control procedures.

Conditioned culture media containing extracellular particulate fractions, membrane-associated proteins, and extracellular structural components were collected and subjected to sequential clarification, concentration, and enrichment procedures designed to reduce cellular debris and non-structured particulate material while preserving membrane-associated extracellular fractions. The resulting preparation consisted of a processed phospholipoproteic secretome-derived formulation enriched in extracellular lipid–protein components and membrane-associated structural proteins [10]. The preparation should not be interpreted as a purified exosome product or as a complete reconstruction of the dermal cellular microenvironment; rather, it represents a processed extracellular lipid–protein preparation enriched in phospholipoproteic and membrane-associated structural components.

Following collection, the secretome-derived material underwent controlled processing procedures, including ultrafiltration-based concentration and purification steps intended to reduce residual soluble contaminants and non-structured aggregates while preserving the extracellular phospholipoproteic fraction [11]. Final preparations were maintained in a sterile isotonic phosphate-buffered aqueous solution composed of 137 mM NaCl, 2.7 mM KCl, 10 mM Na_2_HPO_4_, and 1.8 mM KH_2_PO_4_, adjusted to pH 7.2–7.4. The buffer was prepared using sterile nuclease-free water, filtered through a 0.22 μm membrane under aseptic conditions, and stored at 4 °C until use. For cryopreserved preparations, the same buffered system was supplemented with a cryoprotective excipient mixture suitable for low-temperature storage. Lyophilized samples were reconstituted in the same sterile buffer immediately before downstream proteomic and live-cell kinetic analyses. All preparations were equilibrated under controlled laboratory conditions before sample processing or cellular exposure [12]. The overall preparation, processing, and analytical workflow used throughout the study is summarized in Figure 1.

Representative compositional characteristics of the processed phospholipoproteic formulation are summarized in Table 1.

The processed phospholipoproteic formulation contained a conserved extracellular lipid–protein profile identified by label-free quantitative proteomics. The compositional classes shown in Table 1 are intended to describe the representative architecture of the formulation and should not be interpreted as absolute mass fractions or purified molecular categories, or absolute quantitative concentrations.

### 2.2. Processing Conditions

To evaluate compositional stability under different storage and processing conditions, the processed phospholipoproteic secretome-derived formulation was subjected to four experimental conditions in technical triplicates: (i) fresh formulation (reference condition), (ii) concentrated formulation obtained by tangential-flow ultrafiltration, (iii) cryopreserved formulation stored at −80 °C in cryoprotective buffer, and (iv) lyophilized formulation stored at 4 °C following controlled freeze-drying procedures [13]. Each condition was analyzed in technical triplicate for proteomic characterization. Live-cell kinetic analyses were conducted using independent replicate wells, as described below. The replicate structure was defined before data acquisition and applied consistently across all processing conditions.

All samples were processed under standardized aseptic laboratory conditions and analyzed within predefined stability intervals [14]. The study was designed to compare the relative preservation of the proteomic profile and short-term ex vivo cellular compatibility across processing conditions. It was not designed to evaluate long-term storage performance beyond the predefined analytical intervals or to establish functional biological activity in tissue-level barrier models.

### 2.3. Proteomic Characterization

Bottom-up label-free quantitative proteomic analyses were performed using a timsTOF pro mass spectrometer (Bruker Daltonics GmbH & Co. KG, Bremen, Germany) coupled to an Evosep One liquid chromatography system (Evosep Biosystems, Odense, Denmark) [15]. Protein extracts were reduced, alkylated, and digested using sequencing-grade trypsin (Promega Corporation, Madison, WI, USA). Peptide separation was performed using a 44 min chromatographic gradient, and data acquisition was conducted in data-independent acquisition parallel accumulation–serial fragmentation (dia-PASEF) mode [16].

Raw spectral data were analyzed against the Homo sapiens UniProt database (reviewed entries, May 2026 release) using MaxQuant (v2.4 or later) with match-between-runs enabled. Label-free quantification (LFQ) was performed using IonQuant or equivalent computational workflows [17]. False discovery rate (FDR) thresholds were controlled at <1% at both peptide and protein levels using decoy-based approaches. Protein identification criteria included detection of at least two unique peptides per protein. Oxidation of methionine and N-terminal acetylation were considered variable modifications, while carbamidomethylation of cysteine was considered a fixed modification.

Proteomic data processing was performed using normalized LFQ intensity values after exclusion of proteins that did not meet the predefined identification criteria. Protein intensities were log2-transformed before comparative statistical analysis. Replicate-level analyses were conducted within each processing condition, and batch structure was reviewed before downstream multivariate analysis. Missing values were inspected prior to statistical testing and handled according to predefined filtering rules to reduce the risk of interpreting sporadic identifications as biologically meaningful differences. Proteins retained for comparative analysis were required to meet identification and quantification thresholds across replicate groups. Differential expression analyses were performed using normalized log2 LFQ intensities, with fold-change thresholds and adjusted statistical significance criteria applied consistently across pairwise comparisons.

**Figure 2:**
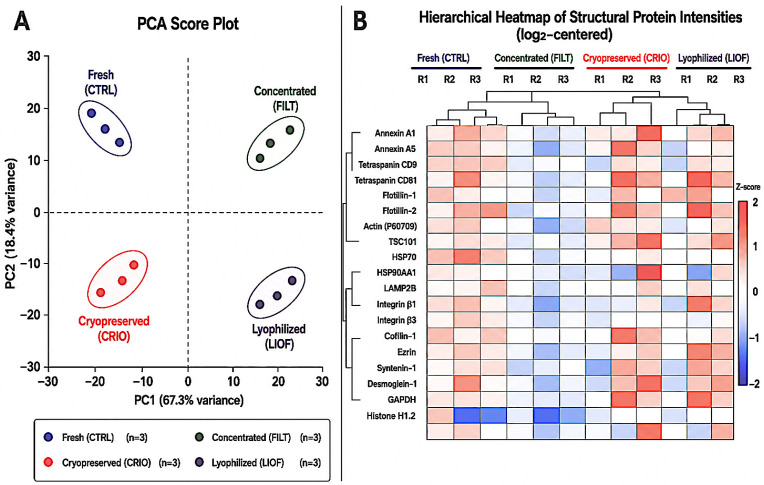
Principal component analysis (PCA) and hierarchical heatmap of representative structural protein intensities across processing conditions. (**A**) PCA score plot generated from normalized label-free quantitative proteomic datasets showing clustering according to processing condition and high inter-replicate reproducibility across fresh, concentrated, cryopreserved, and lyophilized formulations. Each point represents an analytical replicate within the corresponding processing condition. (**B**) Hierarchical heatmap of representative membrane-associated and extracellular structural proteins based on normalized LFQ intensities. Color scaling reflects relative abundance after Z-score normalization. The analysis illustrates condition-specific clustering while supporting preservation of the overall structural proteomic profile following concentration, cryopreservation, and lyophilization procedures.

**Figure 3:**
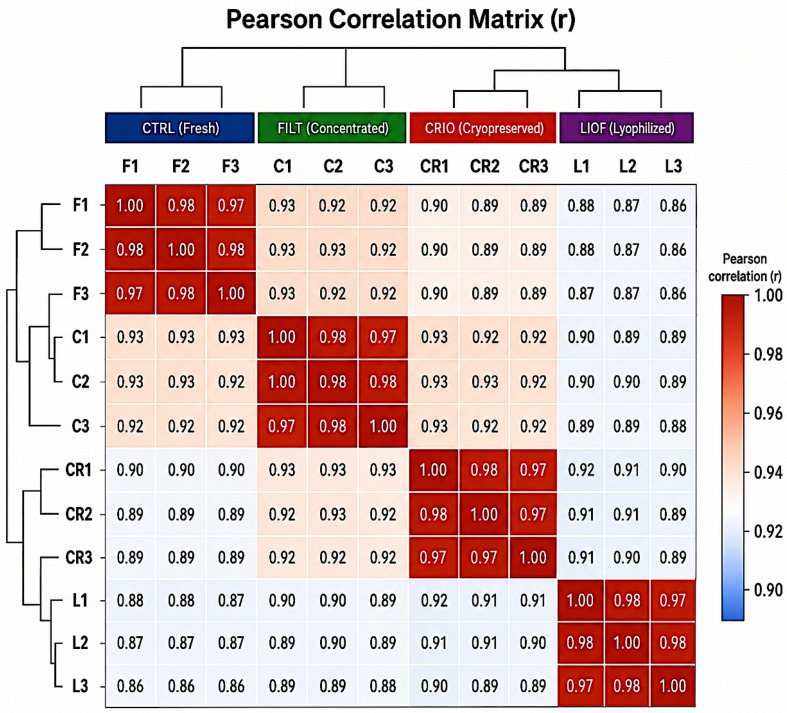
Pearson correlation matrix generated from normalized label-free quantitative proteomic datasets across all processing conditions. Correlation coefficients reflect inter-replicate and inter-condition similarity based on normalized LFQ intensity profiles. Hierarchical clustering demonstrates high inter-replicate consistency within fresh, concentrated, cryopreserved, and lyophilized formulations, supporting analytical reproducibility and preservation of the overall proteomic profile across processing conditions.

**Figure 4:**
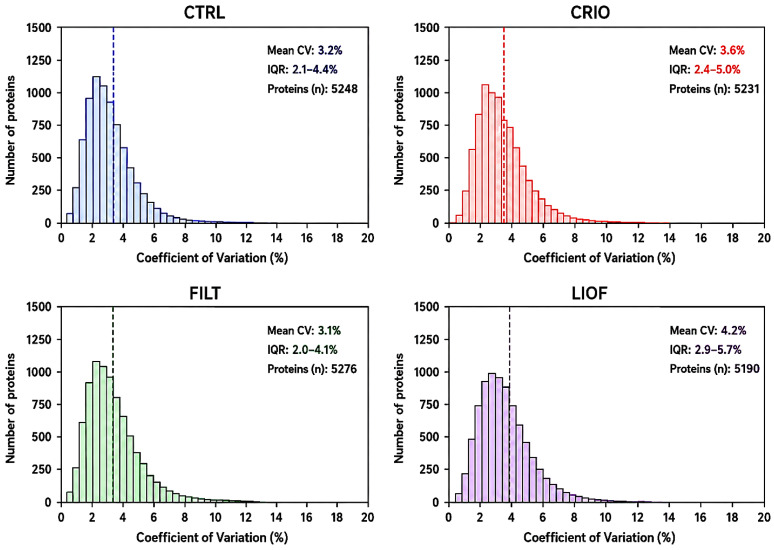
Distribution of coefficients of variation (CV) obtained from normalized label-free quantitative proteomic datasets across processing conditions. Histograms represent protein-level CV distributions within fresh (CTRL), cryopreserved (CRIO), concentrated (FILT), and lyophilized (LIOF) formulations. Dashed vertical lines indicate mean CV values for each condition. Across all experimental groups, most quantified proteins exhibited low CV values, consistent with high analytical reproducibility and preservation of quantitative stability following processing and storage procedures.

**Figure 5:**
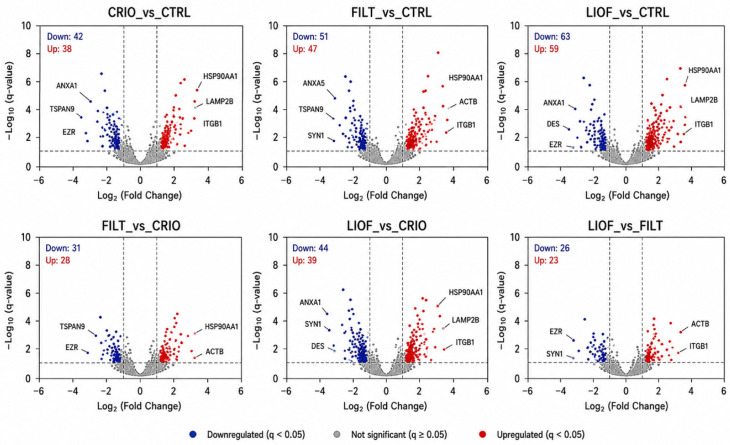
Differential proteomic profiles across processing conditions evaluated by label-free quantitative proteomics. Volcano plots show protein-level pairwise comparisons between cryopreserved (CRIO), concentrated (FILT), lyophilized (LIOF), and fresh control (CTRL) formulations. Red points indicate significantly upregulated proteins, blue points indicate significantly downregulated proteins, and grey points indicate proteins not meeting the predefined differential expression criteria. Across all comparisons, partial divergence was observed while the majority of quantified proteins remained within conserved abundance ranges.

Comparative proteomic analyses included principal component analysis and hierarchical clustering heatmaps (Figure 2), Pearson correlation matrices (Figure 3), coefficient of variation distributions (Figure 4), and differential proteomic profiling by volcano plot analysis (Figure 5). Supplementary proteomic analyses additionally included peptide overlap distributions (Figure S1), protein overlap distributions (Figure S2), hierarchical heatmaps of differentially expressed proteins (Figure S3), and extended volcano plot analyses across all pairwise comparisons (Figure S4). Complete protein identification datasets, differential expression analyses, LFQ intensity matrices, and statistical summaries are provided in Supplementary Tables S1–S4.

Proteomic stability and reproducibility metrics across all processing conditions are summarized in Table 2.

Proteomic analyses were performed using label-free quantitative workflows based on timsTOF Pro dia-PASEF acquisition. Concentrated, cryopreserved, and lyophilized formulations demonstrated preservation of the overall structural proteomic profile relative to the fresh reference condition, with retention of membrane-associated and extracellular structural proteins consistently exceeding 90%.

### 2.4. Short-Term Real-Time Live-Cell Kinetic Profiling

Non-malignant dermal-derived cells were seeded in 96-well plates at optimized densities and allowed to adhere under standard culture conditions. The dermal-derived cells used for kinetic compatibility testing corresponded to research-grade established non-malignant human dermal fibroblast cells, internal study culture code FIC-BX-hDF-IKP-2026-01, provided by Flowinmunocell-Bioexocell Group, Barcelona, Spain, and maintained under standardized in vitro/ex vivo laboratory conditions. These cells were used only as an experimental compatibility model. No primary human skin samples, biopsies, donor-identifiable tissues, or associated clinical data were collected or used for the purposes of this study. The processed phospholipoproteic secretome-derived formulation, normalized according to total protein content, or corresponding vehicle controls, was subsequently added to the cultures as a single-exposure, short-term compatibility assessment. Cellular kinetics were monitored continuously for 48 h using the IncuCyte^®^ S3 live-cell imaging system (Sartorius, Ann Arbor, MI, USA) at 37 °C and 5% CO_2_ [18].

Phase-contrast images were acquired at hourly intervals. Cell confluence was quantified automatically using IncuCyte analysis software (2025–2026 release algorithms) [19]. Morphological assessment was performed throughout the experimental period to evaluate the presence of cytotoxic features, including cellular detachment, blebbing, or abnormal rounding [20]. Experiments were conducted using biological triplicates with technical replicate wells within each experimental condition.

**Figure 6:**
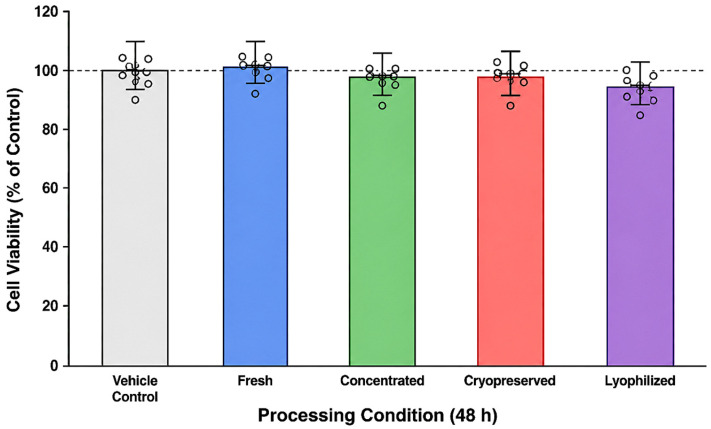
Cell viability of non-malignant dermal-derived cells following 48 h exposure to vehicle control or processed phospholipoproteic secretome-derived formulations under fresh, concentrated, cryopreserved, and lyophilized conditions. Viability values remained comparable across all experimental groups without evidence of detectable acute cytotoxicity under the conditions tested. Data are presented as mean ± SD from independent replicate wells.

**Figure 7:**
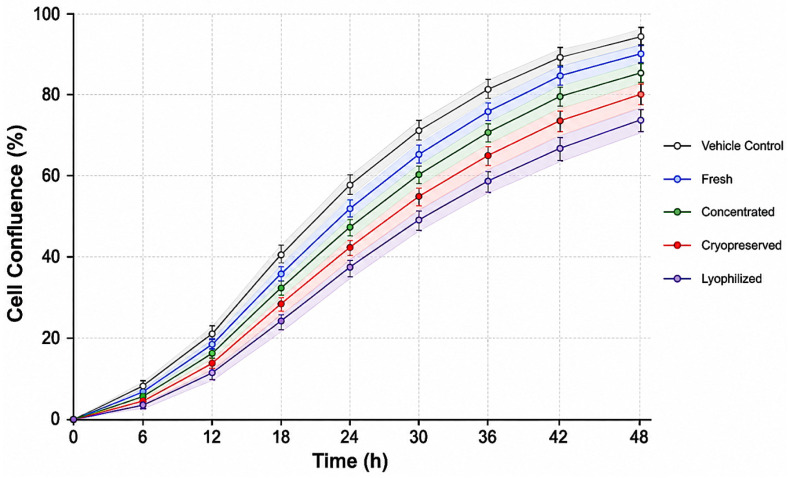
Real-time confluence kinetics of non-malignant dermal-derived cells exposed to vehicle control or processed phospholipoproteic secretome-derived formulations under fresh, concentrated, cryopreserved, and lyophilized conditions. Confluence was monitored continuously by label-free live-cell imaging over 48 h. All experimental groups demonstrated comparable short-term kinetic profiles without evidence of acute proliferative suppression or detectable cytotoxic morphology under the conditions tested. Data are presented as mean ± SD from independent replicate wells.

Representative confluence kinetics are shown in Figure 6, while viability endpoint analyses are presented in Figure 7. Quantitative kinetic compatibility metrics are summarized in Table 3.

Real-time live-cell kinetic imaging analyses were performed using the IncuCyte^®^ S3 platform under continuous monitoring conditions over a 48 h experimental period. Across all formulations, cell viability remained above 92%, with confluence kinetics remaining comparable to vehicle-treated controls and without evidence of short-term proliferative suppression or abnormal kinetic behavior.

### 2.5. Data Integration and Quality Control

Proteomic and live-cell kinetic datasets were systematically documented, cross-referenced, and integrated through internal quality control procedures designed to evaluate analytical consistency, traceability, and inter-condition comparability [21]. The live-cell kinetic component was interpreted according to established IncuCyte-based approaches for the dynamic assessment of live-cell behavior in two-dimensional cultures [22].

### 2.6. Statistical Analysis

Statistical analyses were performed using GraphPad Prism (v10.0 or later) and R statistical software (v4.4 or later). Data are presented as mean ± standard deviation (SD), unless otherwise indicated. Reproducibility across replicates was evaluated using coefficients of variation (CV) and Pearson correlation analyses. Proteomic LFQ intensity values were normalized and log2-transformed prior to comparative analysis. Differential expression analyses were performed using predefined fold-change criteria together with adjusted statistical significance thresholds where applicable, in order to account for multiple comparisons in proteomic datasets. Inter-group comparisons for live-cell kinetic and viability analyses were performed using one-way ANOVA followed by Tukey’s post hoc test or unpaired two-tailed Student’s *t*-tests, as appropriate. Statistical significance for conventional cellular assays was defined as *p* < 0.05. All analytical procedures and acceptance criteria were defined prior to data processing [23].

### 2.7. Quality Control and Acceptance Criteria

Predefined study acceptance criteria included: (i) protein identification with at least two unique peptides and FDR < 1%; (ii) reproducible LFQ intensity profiles across replicates with global CV values within predefined analytical thresholds; (iii) cell viability exceeding 92% at 48 h; and (iv) absence of sustained divergence in confluence kinetics relative to vehicle-treated controls together with absence of morphological evidence of cytotoxicity. Any deviations from predefined criteria were documented and reviewed during data analysis [24].

These acceptance criteria were defined for analytical reproducibility, proteomic stability, and short-term ex vivo compatibility. They were not intended to establish functional skin barrier activity, transepithelial resistance, permeability modulation, tight junction remodeling, lipid raft localization, apoptosis regulation, or long-term tissue-level biological effects.

## 3. Results

### 3.1. Proteomic Fingerprint and Structural Protein Conservation

Bottom-up label-free quantitative proteomic analysis performed using timsTOF Pro instrumentation coupled to dia-PASEF acquisition identified a total of 574 human proteins across all experimental conditions following predefined analytical quality filtering criteria, including false discovery rate (FDR) < 1% at both peptide and protein levels and a minimum of two unique peptides per protein (Table 2, Tables S1 and S3) [25]. Comparative analyses demonstrated preservation of the overall structural proteomic profile across concentrated, cryopreserved, and lyophilized formulations relative to the fresh reference condition. Quantitative retention of membrane-associated and extracellular structural proteins exceeded 90% across all evaluated processing conditions (Table 2) [26].

Peptide- and protein-level overlap analyses demonstrated substantial conservation of identified molecular components across all processing conditions. Venn analyses of peptide overlap distributions revealed preservation of the majority of peptide signatures between fresh, concentrated, cryopreserved, and lyophilized formulations (Figure S1). Similarly, protein overlap analyses demonstrated broad conservation of the identified structural proteomic profile across all experimental groups (Figure S2). Complete protein identification datasets are provided in Supplementary Table S1.

Consistently, Pearson correlation analyses demonstrated strong inter-replicate similarity across all experimental groups, supporting preservation of analytical reproducibility following concentration, cryopreservation, and lyophilization procedures (Figure 3).

Label-free quantification (LFQ) analyses demonstrated high inter-replicate reproducibility, with mean coefficients of variation ranging from 3.0% to 4.5% depending on the experimental condition (Figure 4; Supplementary Table S4), remaining within accepted analytical thresholds for high-resolution dia-PASEF workflows on timsTOF platforms [27]. Principal component analysis (PCA) demonstrated distinct but internally coherent clustering according to processing condition while preserving the overall structural proteomic organization of the datasets (Figure 2A) [28].

Hierarchical clustering heatmaps generated from normalized LFQ datasets demonstrated preservation of representative membrane-associated and extracellular structural proteins across all formulations (Figure 2B). Extended hierarchical clustering analyses of differentially expressed proteins (DEPs) further demonstrated partial proteomic divergence between processing conditions while maintaining conservation of the overall structural proteomic architecture (Figure S3) [29].

Differential expression analyses identified limited compositional divergence between processing conditions relative to the fresh reference formulation. Pairwise volcano plot analyses demonstrated that the majority of quantified proteins remained within conserved abundance ranges despite detectable differential expression patterns following concentration, cryopreservation, and lyophilization procedures (Figure 5 and Figure S4).

The highest degree of divergence was observed in comparisons involving lyophilized formulations, whereas cryopreserved and concentrated formulations demonstrated comparatively lower levels of differential expression (Supplementary Table S2). Data preprocessing, normalization, and LFQ were performed using MaxQuant workflows with match-between-runs and IonQuant-assisted analysis to improve inter-run comparability and peptide alignment [30].

Additional analytical quality metrics, including replicate consistency, sequence coverage distributions, peptide-spectrum matching stability, and inter-condition reproducibility statistics, remained within predefined acceptance criteria throughout all experimental batches (Supplementary Table S4) [31,32]. Overall, the observed analytical reproducibility and preservation of the structural proteomic profile were consistent with current high-throughput membrane-associated proteomic workflows performed using timsTOF-based acquisition platforms [33].

### 3.2. Short-Term Real-Time Kinetic Profiling and Ex Vivo Compatibility

Real-time live-cell kinetic imaging performed using the IncuCyte^®^ S3 platform over a 48 h observation period demonstrated stable short-term confluence kinetics and cellular viability exceeding 92% in non-malignant dermal-derived cells exposed to the processed phospholipoproteic secretome-derived formulation under all evaluated processing conditions (Figure 6 and Figure 7; Table 3) [34].

Confluence kinetics remained comparable to vehicle-treated controls throughout the experimental period, with maximum divergence values below 7% and without evidence of short-term proliferative suppression or abnormal kinetic behavior under the conditions analyzed (Figure 7; Table 3) [35].

Intra-experimental coefficients of variation for confluence measurements remained below 10% across all experimental groups, supporting high reproducibility of the live-cell kinetic analyses (Table 3 and Table S4) [36]. Continuous phase-contrast monitoring demonstrated preservation of normal adherent cellular morphology without evidence of cytotoxic alterations, including membrane blebbing, abnormal rounding, or detachment events during the experimental observation period.

Representative real-time kinetic profiles demonstrated comparable 48 h confluence trajectories between fresh, concentrated, cryopreserved, and lyophilized formulations relative to vehicle-treated controls (Figure 7). Endpoint viability analyses similarly demonstrated preservation of short-term cellular viability across all experimental groups without detectable evidence of acute cytotoxicity following exposure to processed formulations subjected to concentration, cryopreservation, or lyophilization procedures (Figure 6).

Collectively, these findings support short-term ex vivo cellular compatibility and stable kinetic behavior across the processing and storage conditions analyzed. The evaluated phospholipoproteic secretome-derived formulation maintained reproducible 48 h kinetic profiles and preserved cellular viability without detectable acute proliferative disruption or cytotoxic morphology under the experimental conditions tested. These results should be interpreted as an acute ex vivo compatibility assessment and not as evidence of long-term cellular safety, repeated-exposure compatibility, apoptosis modulation, or functional barrier activity.

### 3.3. Functional Annotation of Processing-Associated Proteomic Changes

To provide biological context for the quantitative proteomic dataset, the identified proteins and differentially expressed subsets were interpreted according to predominant functional classes. The overall identified proteomic profile included proteins associated with extracellular structural organization, membrane-associated processes, cytoskeletal architecture, phospholipid binding, vesicle-associated transport, protein folding, and stress-response pathways. These functional categories were consistent with the extracellular lipid–protein and membrane-associated nature of the evaluated preparation. The relevance of membrane-associated organization is consistent with broader evidence showing that membrane microdomains represent structured lipid–protein domains with important functional and biophysical roles in cellular systems [37].

Processing-associated divergence was most evident in the lyophilized condition, which showed the highest number of differentially expressed proteins relative to the fresh reference formulation. The altered protein subsets included categories related to cytoskeletal-associated organization, membrane scaffolding, protein folding or stress-response processes, and extracellular structural components. In contrast, concentrated and cryopreserved formulations showed comparatively lower divergence, consistent with greater preservation of the global proteomic profile relative to the fresh reference condition.

These findings indicate that processing procedures did not affect all protein classes uniformly. Lyophilization was associated with the greatest relative compositional shift, whereas concentration and cryopreservation preserved a proteomic profile more closely aligned with the fresh reference condition. However, the biological significance of these processing-associated differences remains inferential. The present analysis contextualizes compositional changes at the protein-class level but does not establish functional consequences in barrier activity, membrane incorporation, lipid raft engagement, apoptosis regulation, or long-term cellular behavior. This interpretation is also aligned with previous ex vivo traceability frameworks for phospholipoproteomic formulations, in which analytical characterization and functional interpretation were separated from clinical exposure or therapeutic claims [38].

## 4. Discussion

The present study provides a quantitative analytical characterization of a processed phospholipoproteic secretome-derived formulation across fresh, concentrated, cryopreserved, and lyophilized conditions. The principal finding is that the evaluated preparation maintained a largely conserved proteomic profile and high analytical reproducibility across processing conditions while preserving short-term ex vivo cellular compatibility in non-malignant dermal-derived cells during the 48 h observation period. Label-free quantitative proteomic analyses performed using timsTOF Pro dia-PASEF workflows identified 574 human proteins across all experimental conditions, with retention of membrane-associated and extracellular structural proteins exceeding 90% relative to the fresh reference formulation (Table 2). Quantitative reproducibility metrics, including coefficients of variation and inter-replicate Pearson correlations, remained within acceptable analytical ranges (Figure 3 and Figure 4), supporting consistency of the proteomic datasets generated across the evaluated conditions.

These findings indicate that the formulation maintained compositional and analytical stability under defined processing and storage procedures that may otherwise affect extracellular lipid–protein assemblies. Concentration, cryopreservation, and lyophilization can potentially alter relative protein abundance, reduce recovery of selected extracellular structural components, or modify the representation of membrane-associated proteins. In this context, the preservation of the majority of the identified proteomic profile across the evaluated conditions provides a useful analytical basis for comparing processed secretome-derived phospholipoproteic preparations. However, these findings should be interpreted as evidence of compositional conservation and analytical reproducibility, not as direct evidence of functional biological activity.

The formulation evaluated in the present study was derived from processed cell-culture secretome preparations enriched in extracellular lipid–protein and membrane-associated structural components. Preparations of this type are compositionally heterogeneous and may contain soluble proteins, extracellular structural proteins, lipid-associated proteins, membrane-associated components, and particulate extracellular fractions. This heterogeneity is consistent with current evidence showing that extracellular vesicle-related preparations and extracellular particles contain complex and partially overlapping molecular repertoires, making compositional reassessment, proteomic characterization, and standardized analytical interpretation essential [39,40]. This heterogeneity makes quantitative characterization essential, particularly when the material is subjected to processing steps such as ultrafiltration-based concentration, freezing, and lyophilization. The present dataset therefore contributes primarily to the analytical definition of the preparation rather than to the demonstration of a specific mechanism of action.

A relevant observation was that lyophilized formulations showed the highest degree of proteomic divergence relative to the fresh reference condition, whereas concentrated and cryopreserved formulations demonstrated comparatively lower levels of differential expression. This suggests that processing procedures did not affect all compositional classes uniformly. The protein subsets most affected by processing appeared to include categories related to cytoskeletal-associated organization, membrane-associated scaffolding, protein folding or stress-response processes, and extracellular structural components. Although the majority of quantified proteins remained within conserved abundance ranges, the higher divergence observed after lyophilization indicates that freeze-drying may impose greater compositional stress on extracellular lipid–protein assemblies than concentration or cryopreservation. The biological significance of these changes remains inferential and should be further evaluated using dedicated functional assays.

The live-cell kinetic component of the study provides complementary evidence of short-term ex vivo compatibility. Real-time imaging using the IncuCyte^®^ S3 platform showed that exposure of non-malignant dermal-derived cells to the evaluated formulations did not produce detectable acute cytotoxic morphology, abnormal rounding, blebbing, detachment, or short-term proliferative suppression during the 48 h observation period (Figure 6 and Figure 7; Table 3). Cell viability remained above 92% across all evaluated formulations, and confluence trajectories remained broadly comparable to vehicle-treated controls. These results support the interpretation that the evaluated processing conditions did not generate overt acute cytotoxic effects detectable in this experimental model.

Nevertheless, the biological interpretation of the live-cell findings must remain limited. A 48 h single-exposure IncuCyte assay is suitable for evaluating acute cellular compatibility and overt cytotoxicity, but it does not establish long-term compatibility, repeated-exposure tolerance, apoptosis modulation, chronic proliferative behavior, adaptive cellular responses, or tissue-level biological effects. Therefore, the absence of acute cytotoxicity observed in this study should not be interpreted as evidence of long-term safety or functional efficacy. Future studies should include 7–10 day observation periods, repeated exposure models, apoptosis assays, proliferation markers, and additional dermal cell systems, including primary fibroblasts, keratinocytes, and endothelial cells. Expanded cellular and tissue-context models are increasingly used to evaluate extracellular vesicle-related systems, tumor-microenvironment mimetics, organoid-derived extracellular platforms, and extracellular vesicle-based therapeutic strategies, emphasizing the need to interpret short-term compatibility assays within application-specific biological contexts [41,42,43,44].

A central point in interpreting the present study is the distinction between compositional stability and functional barrier validation. Although the identified proteomic profile includes membrane-associated and extracellular structural components, the study did not directly evaluate transepithelial electrical resistance, paracellular permeability, tight junction remodeling, lipid organization, membrane incorporation, lipid raft localization, or supramolecular membrane interactions. Consequently, the present findings do not demonstrate functional skin barrier restoration or direct modulation of membrane organization. Rather, they provide a compositional and short-term ex vivo compatibility framework that may support the design of future mechanistic studies. This distinction is consistent with extracellular vesicle reporting recommendations and with the need to differentiate analytical characterization from functional or therapeutic interpretation in complex extracellular preparations [45].

This distinction is important because dermal barrier function is a complex tissue-level property involving epithelial organization, lipid lamellae, tight junction integrity, extracellular matrix dynamics, immune signaling, and mechanical stress responses. The use of a single non-malignant dermal-derived cell model under controlled ex vivo conditions cannot reproduce the full structural and biochemical complexity of native skin tissue. Accordingly, future functional validation should include TEER measurements, permeability assays, tight junction markers such as claudins, occludin, and ZO-1, lipid organization markers, membrane-localization studies, and mechanically stressed or reconstructed skin models. Advanced biophysical approaches, including super-resolution microscopy, fluorescence recovery after photobleaching, single-particle tracking, and membrane fluidity measurements, may also help determine whether processed phospholipoproteic preparations interact spatially with membrane-associated domains. In parallel, studies on exosome-associated disease progression, extracellular vesicle biomarkers, drug-delivery applications, and immune-context extracellular vesicle biology further illustrate the importance of defining whether extracellular lipid–protein materials act as analytical preparations, delivery systems, biomarkers, or functionally active biological platforms [46,47,48,49,50,51].

The study also has limitations related to source characterization and comparative benchmarking. Although the evaluated formulation was analytically characterized by label-free quantitative proteomics, further work should compare its proteomic profile against well-characterized extracellular vesicle preparations, dermal fibroblast secretomes, keratinocyte-derived secretomes, endothelial cell-derived secretomes, and publicly available extracellular particle or secretome datasets. Such comparative analyses would help position the formulation within the broader field of extracellular lipid–protein preparations and clarify whether its compositional profile is distinct from, partially overlapping with, or functionally comparable to previously characterized secretome-derived systems. For skin-oriented development, extracellular vesicle studies in wound-healing and regenerative models, together with advanced membrane-organization imaging approaches and mesenchymal-stem-cell-derived exosome studies, provide relevant comparators for future evaluation of spatial membrane interactions, dermal compatibility, and tissue-level functional relevance [52,53,54].

In summary, the present study demonstrates that the evaluated processed phospholipoproteic secretome-derived formulation maintained a largely conserved proteomic profile, reproducible analytical behavior, and short-term ex vivo cellular compatibility across defined processing and storage conditions. The findings support the use of label-free quantitative proteomics combined with real-time live-cell kinetic imaging as an analytical framework for evaluating processed extracellular phospholipoproteic preparations. However, the data should be interpreted strictly as evidence of compositional stability, analytical reproducibility, and acute ex vivo compatibility. Functional barrier activity, membrane incorporation, lipid raft engagement, repeated-exposure effects, apoptosis modulation, and long-term tissue-level biological responses remain to be addressed in dedicated future studies [55].

## 5. Conclusions

This study demonstrates that the evaluated processed phospholipoproteic secretome-derived formulation maintained a largely conserved proteomic profile across fresh, concentrated, cryopreserved, and lyophilized conditions. Label-free quantitative proteomics showed preservation of the majority of identified membrane-associated and extracellular structural proteins, with low coefficients of variation and consistent inter-replicate correlations across processing conditions.

Real-time live-cell kinetic imaging further showed that exposure of non-malignant dermal-derived cells to the evaluated formulations did not produce detectable acute cytotoxicity, short-term proliferative suppression, abnormal morphology, blebbing, or detachment during the 48 h observation period. These findings support short-term ex vivo cellular compatibility under the experimental conditions analyzed.

The study provides an analytical framework for evaluating compositional stability and short-term ex vivo compatibility of processed phospholipoproteic secretome-derived preparations. However, the findings do not establish functional skin barrier activity, membrane incorporation, lipid raft engagement, tight junction modulation, repeated-exposure effects, apoptosis regulation, or long-term tissue-level responses. Future studies using TEER, paracellular permeability assays, tight junction markers, lipid organization markers, longer-term kinetic monitoring, repeated-exposure models, and primary dermal cell systems will be required to define the functional relevance of these preparations in skin barrier-oriented experimental models.

## Figures and Tables

**Table 1:** Representative compositional architecture of the processed phospholipoproteic secretome-derived formulation. The categories shown in Table 1 represent compositional classes inferred from the analytical characterization workflow and are not intended to indicate absolute quantitative concentrations of each component class.

Component	Functional Role	Relative Proportion
Phospholipid-associated fraction	Membrane-associated structural organization	Predominant
Membrane-associated proteins	Structural and extracellular compatibility	Conserved proteomic fraction
Extracellular structural proteins	Organization of extracellular lipid–protein assemblies	Moderate abundance
Cytoskeletal-associated proteins	Structural compatibility and membrane-associated interactions	Trace–moderate abundance
Apolipoprotein-associated components	Lipid-associated stabilization	Trace abundance
Annexin-associated proteins	Membrane interaction and phospholipid-binding functions	Trace abundance
Extracellular matrix-associated proteins	Structural extracellular organization	Variable abundance

**Table 2:** Proteomic stability and reproducibility metrics across processing conditions of the processed phospholipoproteic secretome-derived formulation.

Parameter	Fresh (Reference)	Concentrated	Cryopreserved	Lyophilized
Total proteins identified	574	562	545	533
Structural protein retention	100%	>92%	>91%	>90%
Mean coefficient of variation (CV)	3.1%	3.3%	3.0%	4.5%
Inter-replicate Pearson correlation	>0.97	>0.97	>0.96	>0.95
Differentially expressed proteins (DEPs) vs. fresh reference	—	68	49	129
Global proteomic profile conservation:	Reference profile	Preserved	Preserved	Partially preserved
LFQ reproducibility	High	High	High	High

**Table 3:** Real-time kinetic compatibility metrics of non-malignant dermal-derived cells exposed to processed phospholipoproteic secretome-derived formulations under different processing conditions.

Parameter	Vehicle Control	Fresh	Concentrated	Cryopreserved	Lyophilized
Cell viability at 48 h	100 ± 5%	98 ± 7%	98 ± 6%	97 ± 6%	96 ± 7%
Maximum confluence divergence from vehicle	—	<5%	<5%	<6%	<7%
Mean intra-experimental CV	<10%	<9%	<9%	<8%	<10%
Morphological alterations	None detected	None detected	None detected	None detected	None detected
Evidence of blebbing	Absent	Absent	Absent	Absent	Absent
Evidence of detachment	Absent	Absent	Absent	Absent	Absent
Evidence of proliferative suppression	Absent	Absent	Absent	Absent	Absent
Overall kinetic compatibility	Reference profile	Preserved	Preserved	Preserved	Preserved

## Data Availability

The data supporting the findings of this study are available within the article and its Supplementary Materials. Additional data generated and analyzed during the current study, including processed proteomic matrices and live-cell kinetic analysis summaries, are available from the corresponding author upon reasonable request.

## Supplementary Materials

The following supporting information can be downloaded at https://www.mdpi.com/article/10.3390/pharmaceutics18070847/s1, Figure S1: Venn diagrams showing peptide overlap distributions across experimental conditions, including blank control (Blanco), fresh control formulation (CTRL), cryopreserved formulation (CRIO), concentrated formulation (FILT), lyophilized formulation (LIOF), and residual fraction (Residuo). Figure S2: Venn diagrams showing protein overlap distributions across experimental conditions, illustrating conservation of the identified structural proteomic profile following concentration, cryopreservation, and lyophilization procedures. Figure S3: Hierarchical heatmaps of differentially expressed proteins (DEPs) identified across pairwise comparisons between processing conditions, including CRIO_vs_CTRL, FILT_vs_CTRL, LIOF_vs_CTRL, FILT_vs_CRIO, LIOF_vs_CRIO, and LIOF_vs_FILT analyses. Figure S4: Extended volcano plot panels showing differential proteomic profiles across all pairwise comparisons between processing conditions following label-free quantitative proteomic analysis. Table S1: Complete protein identification dataset across all processing conditions obtained by timsTOF Pro dia-PASEF label-free quantitative proteomics. Table S2: Differentially expressed proteins (DEPs) identified across pairwise comparisons between fresh, concentrated, cryopreserved, and lyophilized formulations. Table S3: Label-free quantitative (LFQ) intensity matrix across all experimental replicates and processing conditions. Table S4: Statistical summary of quantitative proteomic analyses and live-cell kinetic profiling, including fold-change distributions, adjusted *p*-values, replicate consistency metrics, coefficient of variation distributions, and correlation analyses.

## Abbreviations

Abbreviation	Full Term
CTRL	Fresh control formulation
CRIO	Cryopreserved formulation
FILT	Concentrated formulation
LIOF	Lyophilized formulation
LFQ	Label-Free Quantification
DEP	Differentially Expressed Protein
CV	Coefficient of Variation
PCA	Principal Component Analysis
FDR	False Discovery Rate
dia-PASEF	Data-Independent Acquisition Parallel Accumulation–Serial Fragmentation
timsTOF Pro	Trapped Ion Mobility Spectrometry Time-of-Flight Pro mass spectrometer
SD	Standard Deviation
Lo	Liquid-Ordered phase
Ld	Liquid-Disordered phase
IncuCyte^®^ S3	Real-time live-cell kinetic imaging system
UniProt	Universal Protein Resource database
ANOVA	Analysis of Variance
Z-score	Standardized score normalization metric
PC1	Principal Component 1
PC2	Principal Component 2
