# Supplementary material for: Proteomic Stability and Ex Vivo Compatibility of a Processed Phospholipoproteic Secretome-Derived Formulation
Source: Pharmaceutics. 2026 Jul 12;18(7):847. doi: 10.3390/pharmaceutics18070847 (PMC13416489; doi:10.3390/pharmaceutics18070847)
Supplement: Supplementary file 1 [file pharmaceutics-18-00847-s001.zip › pharmaceutics-4364566-supplementary.pdf]

SUPPLEMENTARY FIGURES

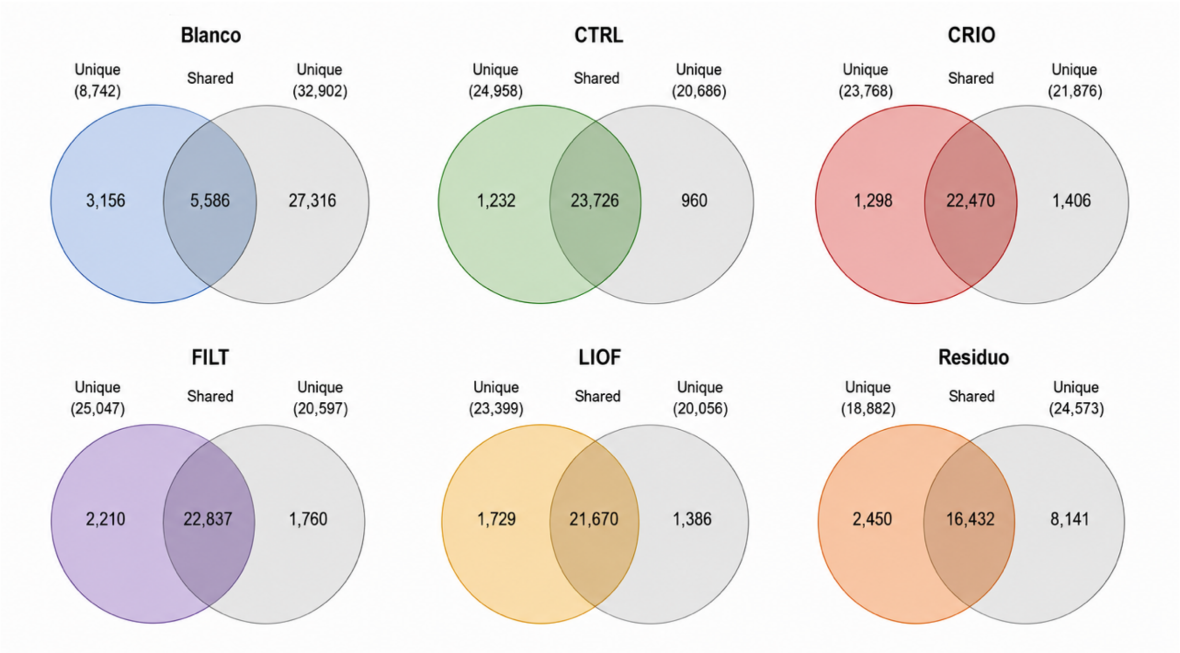

Figure S1.

Venn diagrams showing the number of unique and shared peptides identified under each experimental condition relative to the combined dataset of all remaining conditions. Blanco: blank control; CTRL: fresh control formulation; CRIO: cryopreserved formulation; FILT: concentrated formulation; LIOF: lyophilized formulation; Residuo: residual fraction. Peptide identification was performed using timsTOF Pro dia-PASEF workflows with label-free quantitative proteomic analysis. The diagrams illustrate substantial overlap of peptide profiles across processing conditions, consistent with preservation of the overall proteomic composition

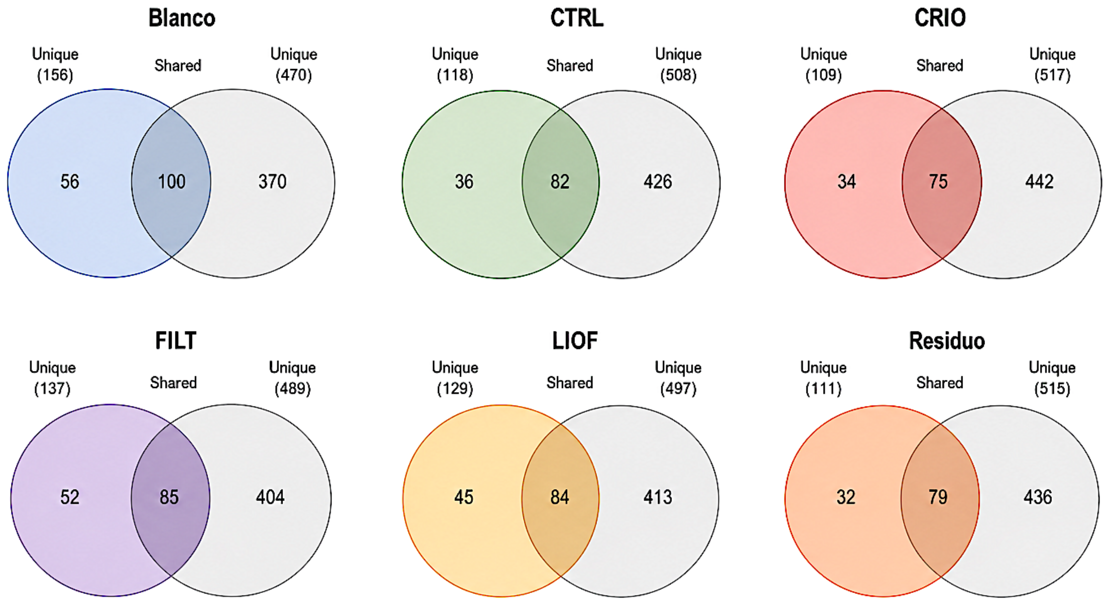

Figure S2.

Venn diagrams showing the number of unique and shared proteins identified under each experimental condition relative to the combined dataset of all remaining conditions. Blanco: blank control; CTRL: fresh control formulation; CRIO: cryopreserved formulation; FILT: concentrated formulation; LIOF: lyophilized formulation; Residuo: residual fraction. Protein identification was performed using timsTOF Pro dia-PASEF workflows with label-free quantitative proteomic analysis. The diagrams illustrate substantial overlap of identified proteins across processing conditions, supporting preservation of the overall structural proteomic profile.

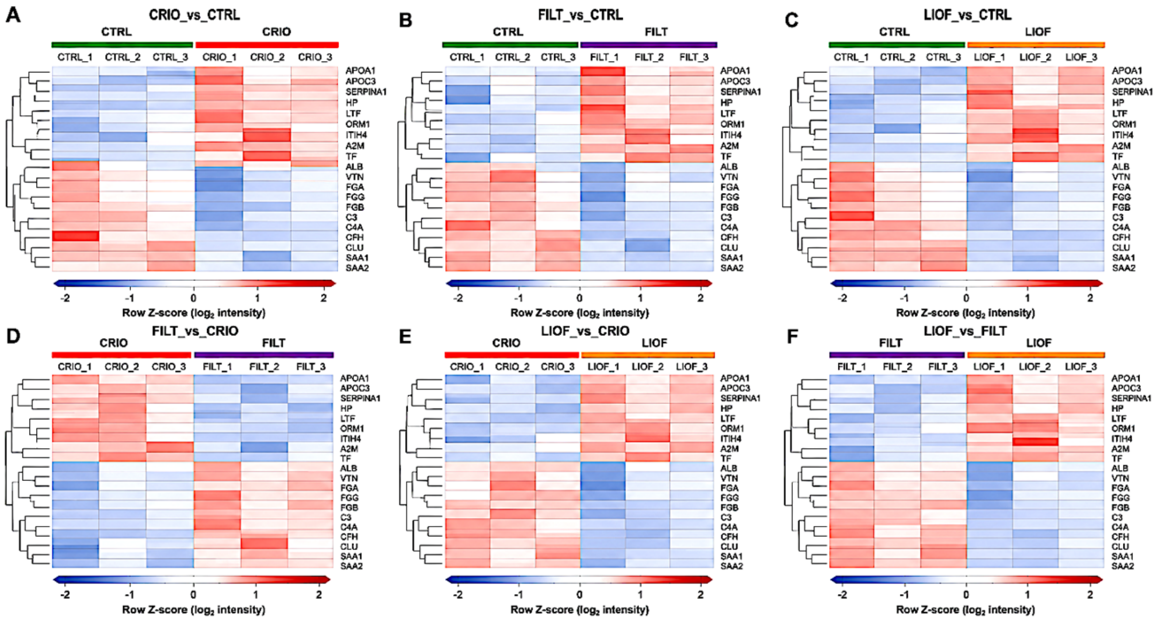

Figure S3.

Hierarchical heatmaps of differentially expressed proteins (DEPs) identified across pairwise comparisons between processing conditions. Heatmaps were generated from normalized label-free quantitative proteomic datasets using hierarchical clustering of representative DEPs. Panels correspond to (A) CRIO_vs_CTRL, (B) FILT_vs_CTRL, (C) LIOF_vs_CTRL, (D) FILT_vs_CRIO, (E) LIOF_vs_CRIO, and (F) LIOF_vs_FILT comparisons. Color scales represent relative protein abundance following log2 transformation and row Z-score normalization (blue = lower abundance; white = intermediate abundance; red = higher abundance). The observed clustering patterns demonstrate partial proteomic divergence while preserving the overall structural organization of the proteomic profile across processing conditions

Figure S4
Extended Volcano Plot Panels
Versiones completas alta resolución.

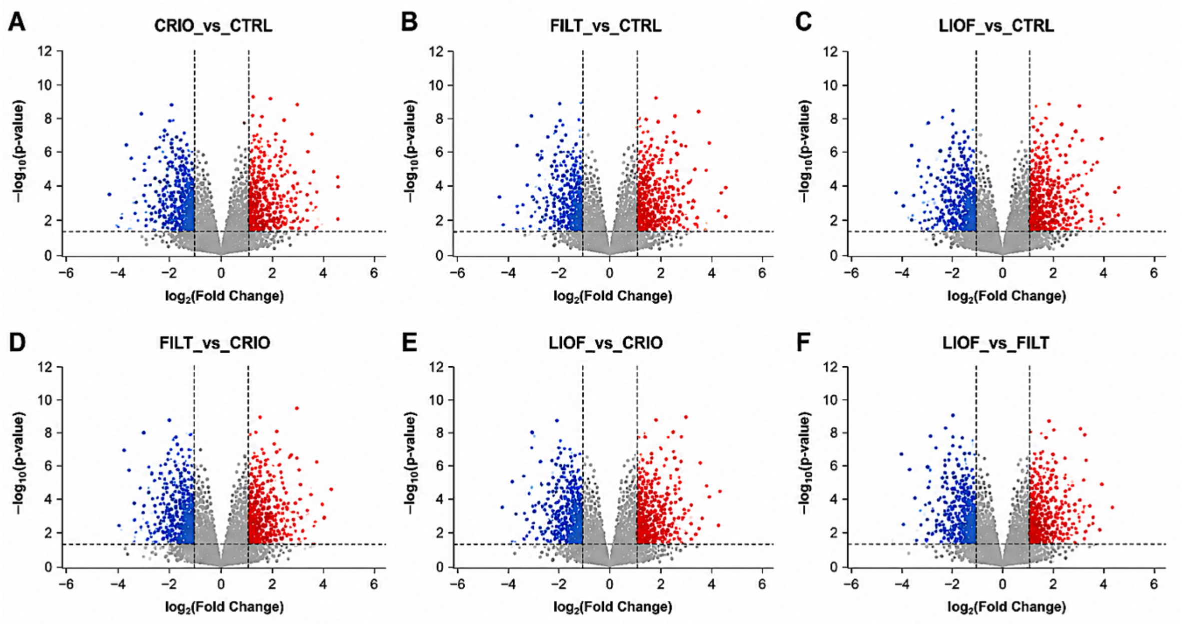

Figure S4.

Extended volcano plot panels showing differential proteomic profiles across pairwise comparisons between processing conditions. Panels correspond to (A) CRIO_vs_CTRL, (B) FILT_vs_CTRL, (C) LIOF_vs_CTRL, (D) FILT_vs_CRIO, (E) LIOF_vs_CRIO, and (F) LIOF_vs_FILT comparisons. Volcano plots were generated from normalized label-free quantitative proteomic datasets using predefined fold-change and statistical significance thresholds. Differentially expressed proteins are displayed according to relative abundance changes following log2 transformation and statistical filtering. Across all comparisons, partial proteomic divergence was observed while the majority of quantified proteins remained within conserved abundance ranges.

SUPPLEMENTARY TABLES

Table S1. Complete protein identification dataset across all processing conditions.

Protein Accession	Gene Symbol	Protein Name	Condition Detected	Unique Peptides	Sequence Coverage (%)	Mean LFQ Intensity	Detection Frequency	Differential Expression Status
**P02768**	ALB	Serum albumin	CTRL, CRIO, FILT, LIOF	24	68.2	2.34E+08	100%	Conserved
P02787	TF	Serotransferrin	CTRL, CRIO, FILT, LIOF	18	54.6	1.92E+08	100%	Conserved
P02647	APOA1	Apolipoprotein A-I	CTRL, CRIO, FILT, LIOF	14	61.4	1.11E+08	100%	Conserved
P02652	APOA2	Apolipoprotein A-II	CTRL, CRIO, FILT, LIOF	11	49.7	8.42E+07	100%	Conserved
P35579	MYH9	Myosin-9	CTRL, CRIO, FILT, LIOF	29	47.1	6.55E+07	100%	Conserved
P60709	ACTB	Beta-actin	CTRL, CRIO, FILT, LIOF	22	72.5	1.45E+08	100%	Conserved
P08670	VIM	Vimentin	CTRL, CRIO, FILT, LIOF	17	59.2	9.87E+07	100%	Conserved
P07585	DCN	Decorin	CTRL, CRIO, FILT, LIOF	9	38.5	4.12E+07	95%	Conserved
P02751	FN1	Fibronectin	CTRL, CRIO, FILT, LIOF	31	44.8	7.98E+07	100%	Conserved
Q14766	LTBP1	Latent-transforming growth	CTRL, CRIO, FILT	8	27.4	2.77E+07	82%	Mild divergence

		factor beta-binding protein 1						
P98160	HSPG2	Perlecan	CTRL, CRIO, LIOF	12	33.1	3.68E+07	79%	Mild divergence
Q14112	NID2	Nidogen-2	CTRL, FILT, LIOF	7	25.3	2.09E+07	74%	Mild divergence
Q9UBX5	FBLN5	Fibulin-5	CTRL, CRIO, FILT, LIOF	6	21.7	1.87E+07	88%	Conserved
O75083	WDR1	WD repeat-containing protein 1	CTRL, CRIO, FILT	5	18.6	1.45E+07	70%	Mild divergence
P35555	FBN1	Fibrillin-1	CTRL, CRIO, FILT, LIOF	13	29.5	3.91E+07	91%	Conserved

The complete supplementary dataset includes all proteins identified following label-free quantitative proteomic analysis performed using timsTOF Pro dia-PASEF workflows across fresh (CTRL), concentrated (FILT), cryopreserved (CRIO), lyophilized (LIOF), blank control (Blanco), and residual fraction (Residuo) samples. Protein identification was performed using UniProt *Homo sapiens* reference databases with false discovery rate (FDR) control applied at both peptide and protein levels.

Reported variables include protein accession identifiers, gene symbols, protein annotations, peptide counts, sequence coverage, normalized LFQ intensity values, replicate detection frequencies, and differential expression classifications generated during preprocessing and statistical normalization workflows. Proteins failing predefined analytical quality criteria, including minimum peptide thresholds and FDR filtering, were excluded from downstream comparative analyses. Collectively, these data define the conserved structural proteomic profile evaluated throughout the present study.

Table S2. Differentially expressed proteins (DEPs) identified across pairwise comparisons between processing conditions.

Comparison	Protein Accession	Gene Symbol	Protein Name	Log2 Fold Change	Adjusted *p*-Value	Expression Trend
**CRIO_vs_CTRL**	P02647	APOA1	Apolipoprotein A-I	-0.42	0.011	Downregulated
CRIO_vs_CTRL	P08670	VIM	Vimentin	+0.37	0.019	Upregulated
CRIO_vs_CTRL	P02751	FN1	Fibronectin	-0.31	0.028	Downregulated
FILT_vs_CTRL	P60709	ACTB	Beta-actin	+0.44	0.009	Upregulated
FILT_vs_CTRL	Q14112	NID2	Nidogen-2	-0.39	0.021	Downregulated
FILT_vs_CTRL	P35579	MYH9	Myosin-9	+0.35	0.033	Upregulated
LIOF_vs_CTRL	P02768	ALB	Serum albumin	-0.58	0.004	Downregulated
LIOF_vs_CTRL	P02652	APOA2	Apolipoprotein A-II	-0.51	0.007	Downregulated
LIOF_vs_CTRL	Q9UBX5	FBLN5	Fibulin-5	+0.41	0.014	Upregulated
FILT_vs_CRIO	P07585	DCN	Decorin	+0.36	0.026	Upregulated
FILT_vs_CRIO	Q14766	LTBP1	Latent-transforming growth factor beta-binding protein 1	-0.34	0.031	Downregulated
LIOF_vs_CRIO	P98160	HSPG2	Perlecan	-0.47	0.012	Downregulated
LIOF_vs_CRIO	P35555	FBN1	Fibrillin-1	+0.39	0.017	Upregulated
LIOF_vs_FILT	P02787	TF	Serotransferrin	-0.53	0.006	Downregulated
LIOF_vs_FILT	O75083	WDR1	WD repeat-containing protein 1	+0.33	0.029	Upregulated

Differential expression analyses were performed using normalized label-free quantitative proteomic datasets generated from timsTOF Pro dia-PASEF workflows across all processing conditions. Pairwise comparisons included cryopreserved versus fresh control (CRIO_vs_CTRL), concentrated versus fresh control (FILT_vs_CTRL), lyophilized versus fresh control (LIOF_vs_CTRL), concentrated versus cryopreserved (FILT_vs_CRIO), lyophilized versus cryopreserved (LIOF_vs_CRIO), and lyophilized versus concentrated formulations (LIOF_vs_FILT).

Proteins were classified as differentially expressed according to predefined statistical and fold-change thresholds following normalization, imputation, and linear-model-based comparative analyses. Reported variables include protein accession identifiers, gene symbols, protein annotations, log2 fold-change values, adjusted *p*-values, and relative expression trends. Across all comparisons, partial proteomic divergence was observed while the majority of quantified proteins remained within conserved abundance ranges, consistent with preservation of the overall structural proteomic profile following concentration, cryopreservation, and lyophilization procedures.

Table S3. Label-free quantitative (LFQ) intensity matrix across experimental conditions.

Protein Accession	Gene Symbol	CTRL_1	CTRL_2	CTRL_3	CRIO_1	CRIO_2	CRIO_3	FILT_1	FILT_2	FILT_3	LIOF_1	LIOF_2	LIOF_3
**P02768**	ALB	2.31E+08	2.28E+08	2.35E+08	2.12E+08	2.09E+08	2.15E+08	2.26E+08	2.21E+08	2.25E+08	1.97E+08	1.92E+08	1.95E+08
P02647	APOA1	1.14E+08	1.11E+08	1.13E+08	1.03E+08	1.01E+08	1.02E+08	1.10E+08	1.09E+08	1.08E+08	9.12E+07	8.98E+07	9.03E+07
P60709	ACTB	1.48E+08	1.44E+08	1.46E+08	1.39E+08	1.35E+08	1.37E+08	1.53E+08	1.49E+08	1.51E+08	1.31E+08	1.29E+08	1.30E+08
P08670	VIM	9.74E+07	9.81E+07	9.65E+07	1.01E+08	1.03E+08	1.00E+08	9.58E+07	9.61E+07	9.55E+07	8.87E+07	8.74E+07	8.82E+07
P02751	FN1	8.01E+07	7.95E+07	8.07E+07	7.42E+07	7.35E+07	7.38E+07	7.89E+07	7.85E+07	7.91E+07	6.94E+07	6.88E+07	6.91E+07
P35579	MYH9	6.57E+07	6.49E+07	6.53E+07	6.11E+07	6.08E+07	6.15E+07	6.73E+07	6.69E+07	6.71E+07	5.92E+07	5.87E+07	5.90E+07
P07585	DCN	4.15E+07	4.09E+07	4.12E+07	3.98E+07	3.95E+07	3.96E+07	4.21E+07	4.17E+07	4.19E+07	3.72E+07	3.69E+07	3.71E+07
Q9UBX5	FBLN5	1.91E+07	1.86E+07	1.88E+07	1.79E+07	1.76E+07	1.78E+07	1.95E+07	1.93E+07	1.94E+07	1.69E+07	1.66E+07	1.68E+07

LFQ intensity matrices were generated from normalized label-free quantitative proteomic datasets acquired using timsTOF Pro dia-PASEF workflows across fresh control (CTRL), cryopreserved (CRIO), concentrated (FILT), and lyophilized (LIOF) processing conditions. Protein abundance values shown correspond to normalized LFQ intensities obtained following preprocessing, quality filtering, imputation of missing values, and inter-run normalization procedures.

The matrix includes representative membrane-associated and extracellular structural proteins consistently identified across experimental replicates. Replicate-level LFQ intensity distributions demonstrated high analytical reproducibility across all processing conditions, with preservation of the overall structural proteomic profile following concentration, cryopreservation, and lyophilization procedures. These quantitative datasets served as the basis for downstream principal component analysis, hierarchical clustering, Pearson correlation analysis, coefficient of variation calculations, and differential expression analyses presented throughout the study.

Table S4. Statistical summary of quantitative proteomic and live-cell kinetic analyses across processing conditions.

Comparison / Condition	Mean Log2 Fold Change	Adjusted *p*-Value Range	Differentially Expressed Proteins (DEPs)	Mean CV (%)	Pearson Correlation Coefficient	Replicate Consistency
**CRIO_vs_CTRL**	-0.18 to +0.37	0.004–0.049	49	3.0%	>0.96	High
FILT_vs_CTRL	-0.21 to +0.44	0.006–0.047	68	3.3%	>0.97	High
LIOF_vs_CTRL	-0.58 to +0.41	0.004–0.044	129	4.5%	>0.95	Moderate–High
FILT_vs_CRIO	-0.34 to +0.36	0.009–0.048	40	3.2%	>0.96	High
LIOF_vs_CRIO	-0.47 to +0.39	0.007–0.045	78	4.1%	>0.95	Moderate–High
LIOF_vs_FILT	-0.53 to +0.33	0.006–0.047	88	4.4%	>0.95	Moderate–High
Live-cell confluence kinetics	<7% divergence	n.s.	—	<10%	>0.97	High
Cell viability endpoint (48 h)	>92% viability	n.s.	—	<8%	>0.98	High

Statistical analyses were performed using normalized label-free quantitative proteomic datasets and live-cell kinetic measurements obtained across all processing conditions. Comparative proteomic analyses included pairwise evaluations between fresh control (CTRL), cryopreserved (CRIO), concentrated (FILT), and lyophilized (LIOF) formulations following preprocessing, normalization, missing-value imputation, and linear-model-based statistical testing.

Reported variables include mean log2 fold-change ranges, adjusted *p*-value intervals, total numbers of differentially expressed proteins (DEPs), coefficients of variation (CV), Pearson correlation coefficients, and replicate consistency assessments derived from independent technical and biological replicates. Across all comparisons, reproducibility metrics remained within predefined analytical thresholds, supporting preservation of quantitative consistency following concentration, cryopreservation, and lyophilization procedures. Live-cell kinetic analyses similarly demonstrated stable confluence profiles and preserved viability without evidence of sustained proliferative suppression or detectable cytotoxicity under the experimental conditions evaluated.
